# Improvement of Skin Wound Healing for Diabetic Mice with Thermosensitive Hydrogel Combined with Insulin Injection

**DOI:** 10.1155/2022/7847011

**Published:** 2022-03-10

**Authors:** Lingling Fang, Haijian Wu, Xiaoyan Li, Jianghua Fang, Yabin Zhu

**Affiliations:** ^1^Endocrinology Department, Ningbo Medical Center Lihuili Hospital, Affiliated to Ningbo University, 1111 Jiangnan Road, Ningbo 315000, China; ^2^School of Medicine, Ningbo University, 818 Fenghua Road, Ningbo 315211, China; ^3^Medical Records and Statistics Office, Ningbo Medical Center Lihuili Hospital, 1111 Jiangnan Road, Ningbo 315000, China; ^4^College of Material Science and Chemical Engineering, Ningbo University of Technology, 201 Fenghua Road, Ningbo 315211, China

## Abstract

Chronic skin wound caused by diabetic disease is very common worldwide. Moreover, there is a shortage of effective curing technology in clinic. In this work, we developed a novel technology using thermosensitive hydrogel on wound top combined with insulin injection. The efficiency and mechanism of this technology were investigated in a diabetic mouse model. Dorsal-paired 8–10 mm diameter wounds were created in 12 mice. The wound healing rate was determined over a 28-day interval in healthy control (Control), control with diabetes (DControl), poloxamer treatment (Pox), and poloxamer plus insulin injection (Poxin) mice. Histological specimens were observed in all samples. Real-time quantitative polymerase chain reaction (qRT-PCR) was performed to measure the relative expression of *α*-smooth muscle actin (*α*-SMA) and transforming growth factor beta 1 (TGF-*β*1) in wound tissues at 7, 14, and 28 days. Compared with DControl animals, those treated with Poxin showed accelerated wound closure and healing rate (*p* < 0.05); expression of both *α*-SMA and TGF-*β*1 was significantly higher than that of the DControl and Pox animals during the first 7 days postoperation, but a significant decrease at day 14. Therefore, we concluded that hydrogel combined with insulin accelerated wound healing. Controlling the glucose level via insulin injection is more beneficial than hydrogel alone for healing chronic wounds, potentially through the increase of *α*-SMA and TGF-*β*1 expression in early phase.

## 1. Introduction

Diabetes mellitus (DM) is a chronic disease and a severe global public health problem. It has been reported that there were 451 million people (age 18–99 years) with diabetes in 2017 worldwide, which is expected to increase to 693 million by 2045 [1]. Moreover, almost half of all people (49.7%) living with diabetes are undiagnosed [1]. Delayed wound healing is an important complication in patients with diabetes due to their macrovascular and microvascular lesions [2]. More than 68 million diabetic individuals develop diabetic wounds worldwide [3]. These wounds also impose a heavy worldwide social, economic, and health burden due to a lack of efficient wound healing agents. About 2.5%–15% of yearly worldwide health budgets are consumed on diabetes mellitus, and diabetic wounds take a major part [3]. Some previous studies [4, 5] have reported that sustained high glucose levels in patients with diabetes impaired the skin cell proliferation and collagen production, both of which impeded wound healing after skin injury. Also, pathological microvascular changes, persistent increment of proinflammatory cytokines, and the absence of growth factors are also associated with delayed wound healing [6, 7].

Wound repair involves complex overlapping processes, including hemostasis, inflammation, cell proliferation, angiogenesis, re-epithelialization, and skin remodeling [8, 9]. During hemostasis, a fibrin plug is formed, which leads platelets to release cytokines and growth factors, such as transforming growth factor-*β* (TGF-*β*), which recruits inflammatory cells to the wound site [10]. In the inflammatory phase, under the chemotaxis of TGF-*β*1 secreted by platelets and the activation of proinflammatory cytokines, interleukin (IL)-1*β*, and tumor necrosis factor-alpha (TNF-*α*), neutrophils migrate to the wound site to remove necrotic tissue, debris, and pathogenic microorganisms [11]. The proliferation phase involves the migration and proliferation of keratinocytes at the wound site and is characterized by increased neovascularization, granulation formation, collagen deposition, and epithelialization [10]. The tissue remodeling phase restores tissue structural integrity and functional competence [12].

With in-depth growth of molecular biology related to wound healing and rapid development of biomaterials for tissue engineering, an increasing number of materials have been studied for wound care. In particular, products in the form of hydrogels have become the important dressings in clinical practice. Poloxamers are nonionic triblock copolymers composed of polyoxyethylene, with strong hydrophilicity at both ends, and polyoxypropylene, with strong hydrophobicity in the middle, which received the patent in 1973. Among these poloxamers, poloxamer 407 is widely employed for drug delivery because it is reported to be nontoxic and can form gels at 25°C at a concentration of 10–20 wt% [13, 14]. It is also favored and often applied by many researchers to prepare temperature-sensitive hydrogels [15, 16]. In our previous work, Poloxamer 407 was also used as hydrogel-like material to assist the repair of defected cornea or skin [17, 18]. Poloxamer 407 has been verified to have good biocompatibility.

On the other hand, insulin therapy is a common method to treat diabetes that involves regulating glucose and lipid metabolism. Insulin also plays an important role in protein synthesis, mitochondrial biogenesis, cell growth, proliferation, differentiation, and migration [19]. Insulin can promote the transition for high-glucose-induced macrophages from M1 to M2 phenotype and modulate inflammation through the reduction of proinflammatory cytokines and the induction of anti-inflammatory cytokines, finally promoting diabetic wound healing [20]. With regard to anti-inflammatory cytokines, transforming growth factor (TGF)-*β*1 plays a crucial role in the inflammatory progression during wound recovery [21]. Until the end of the proliferative phase, TGF-*β*1 induces the expression of*α*-smooth muscle actin (SMA), which promotes the tissue contraction, re-epithelialization, and eventual closure of the wound [22, 23]. Therefore, we supposed that thermosensitive hydrogel Poloxamer 407 combined with insulin injection might be helpful to promote wound healing for diabetes. This research has not yet been reported in the literature.

In this study, we investigated the role of poloxamer and insulin in early and late phases of wound healing progress using streptozotocin (STZ)-induced diabetic mice, with the aim of elucidating the function and biological mechanism of poloxamer and insulin together. Hematoxylin and eosin (HE) staining was used to observe the morphological characteristics of each group of samples at each time point. Immunohistochemistry and qRT-PCR were used to explore the expression of *α*-SMA and TGF-*β*1 at the protein and gene level in different wound healing phases. These results, application of material plus medicine, will provide very important information for our future work about wound healing efficiency and mechanism.

## 2. Materials and Methods

### 2.1. Preparation of Hydrogel

Poloxamer 407 (Beijing Solarbio Science & Technology Co., Ltd., China) was dissolved in deionized cold water to obtain the aqueous solution (Pox) with the concentration of 12 wt% at 4°C. The solution was sterilized by filtration using a disposable filter tip (0.22 *μ*m; Wuxi NEST Biotechnology Co., Ltd., China) in the clean bench. Pox solution will become gel at 25°C from liquid at 4°C.

### 2.2. Establishment of the Diabetic Mice Model

Streptozotocin (STZ; Sigma Co., USA) (70 mg/kg animal) was intraperitoneally injected to Institute of Cancer Research (ICR) mice (five-week-old, male) once every other day for a total of five injections. Venous blood was collected from the tail of each mouse daily to test the blood sugar level. Modeling of diabetes was considered successful when the postprandial blood glucose remained ≥16.7 mmol/L throughout the experimental period, while the general bodyweight of mice reduced from 28.96 ± 1.37 g (normal) to 27.67 ± 0.58 g (diabetes).

The ICR mice used in this experiment were provided by Weitong Lihua Experimental Animal Technology Co., Ltd. (Zhejiang Province, China; animal certificate number: SCXK (Zhejiang) 2018–0001). The procedures for animal handling complied with the principle of animal care in the Animal Laboratory Center, Ningbo University, and the National Health Commission. A body-weight reduction of 20% for experimental animals is defined as a humane endpoint.

### 2.3. Full-Thickness Skin Defects and Treatments in Diabetic Mice

The mice were anesthetized with 1% sodium pentobarbital via intraperitoneal injection, and the hair on the back of the mice was removed by using a shaver after applying a depilatory cream. After disinfecting the skin of the mouse with a 75% alcohol wipe, a hole punch was used to remove the skin at a diameter of 0.8–1.0 cm on the back of each animal to establish the full-thickness skin defect mouse model.

**Figure 1:**
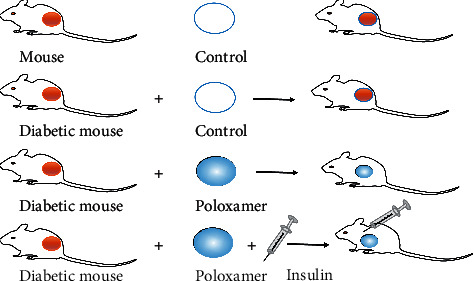
The experimental mice model: control, diabetic mouse (DControl), poloxamer (Pox), and poloxamer plus insulin (Poxin).

The experimental animals were then divided into four groups: normal mice (Control), diabetic mouse (DControl), diabetic mice with poloxamer treatment (Pox), and diabetic mice with poloxamer plus insulin treatment (Poxin) (Figure 1). The mice of the Control and DControl groups were left untreated but directly covered with a transparent film. The mice of Pox group were topically treated with 10 *μ*L poloxamer solution. For the mice of Poxin group, insulin was injected once in the morning and the evening daily at a dose of 5 IU/kg/d, which was prepared from insulin stock solution (40 IU/ml) diluted 40 times with 5% glucose solution to get 1.0 IU/ml for use. At the same time, 10 *μ*L poloxamer solution was applied to the wound top. After that, the blood glucose changes were measured with a blood glucose meter.

All wounds were covered with a transparent film after the treatment. The poloxamer hydrogel was reapplied, and the transparent film was replaced every other day for the first 8 days postoperation. Diabetic mice were individually housed in separate cages to prevent fighting and disturbance of the wounds.

### 2.4. General Observation of Wounds

The wounds in diabetic mice with full-thickness skin defects were monitored every other day by taking photographs, measuring the wound area, and recording the details. The Image-Pro Plus analysis software package (Media Cybernetics, Bethesda, MD) was used to calculate the changes in the wound area and prepare a curve showing the changes in wound area over time (using time as a variable). The percentage of wound area changes was calculated using the following equation: change rate of wound area (%) = wound area at different time points/initial wound area × 100%.

### 2.5. *In Vivo* Tests

Samples of the wound site and surrounding skin tissue were collected on days 7, 14, and 21 after the operation. First, each experimental mouse was anesthetized with 50 mg/kg sodium pentobarbital, followed by removing the newly grown hair on the wound with depilatory cream and a shaver and wiping the wound and surrounding area with 75% alcohol. Next, the full-thickness skin approximately 3 mm along the periphery of the wound was collected. The sample was immediately placed in the embedding agent, optimal cutting temperature compound (OCT; SAKURA, USA), for later histomorphological analyses.

#### 2.5.1. HE Staining

Hematoxylin-eosin (HE) staining was carried out according to our previous work [24]. The samples were sectioned into 5-*μ*m thick sections using a microtome (Leica CM1950, Germany) and soaked in water for 5 s to remove OCT. The sections were subjected to nuclear staining in hematoxylin (H) solution for 7 min, and then the excess hematoxylin was removed by submerging in 1% hydrochloric acid/alcohol solution for 3 s and in 1% ammonia water for 10 s. The sample was then rinsed with water and soaked in 0.5% eosin solution for 3 s to stain the cytoplasm. Excess eosin (E) was removed by three washes in clean water. The tissue slices were hydrated in gradient alcohol solutions (75%, 80%, 95%, 95%, 100%, and 100% in volume ratio) for 2 s each, transparency in dimethylbenzene twice for 3 s each, and then sealed with neutral gum. The morphology of the tissue sections was observed under a light microscope (Olympus CX40, Japan).

#### 2.5.2. Immunohistochemistry Analysis

Immunohistochemistry analysis, based on antigen-antibody reactions, was performed on 5-*μ*m thick sections of skin specimens of control and different treated groups, in order to reveal the localization, quality, and relative quantity of the antigens in the tissues. The procedure was followed from the method described by Feng et al. [18]. In brief, the sample was soaked in water for 5 s to remove OCT reagent. A hydrophobic barrier pen was used to draw circles around the tissue slice on the glass slide. Hydrogen peroxide solution (3% by volume) was dropped onto the slice within the circle followed by incubation at room temperature for 5 min to quench the endogenous peroxidase and prevent false-positive staining. The tissue slices were subsequently washed and blocked in 10% goat serum (by volume) at room temperature for 1 h to eliminate nonspecific staining and improve the accuracy of specific protein staining. The tissue slices were then incubated with *α*-SMA (1 : 250; Boster, China) or TGF-*β*1 (1 : 250; Abcam, China) and washed in phosphate buffered saline (PBS) thrice (3 min each).

Tissue slices were incubated with horseradish peroxidase (HRP)-conjugated goat anti-mouse immunoglobulin secondary antibodies (PV-6002; ZSBiO, China) for 20 min at 37°C and then washed in PBS thrice (3 min each). The tissue slices were developed in 3,3′-diaminobenzidine solution (DAB; ZSBiO, China) at 1 : 20 according to the manufacturer's instructions until brown staining was observed, followed by quickly placing in clean water to stop the color development. The tissue slices were hydrated in gradient alcohol solutions (75%, 80%, 95%, 95%, 100%, and 100% in volume ratio) for 2 s each, transparency in dimethylbenzene twice for 3 s each, and then sealed with neutral gum. The morphology of the skin repair conditions of the tissue sections was observed under a light microscope (Olympus CX40, Japan).

#### 2.5.3. Real-Time Fluorescent Quantitative Polymerase Chain Reaction (qRT-PCR)

All tools for total RNA extraction, including scissors, tweezers, and pestles, were presoaked in diethylpyrocarbonate (DEPC; Solarbio, China) solution overnight, followed by autoclaving before RNA extraction. The frozen skin tissue was removed from liquid nitrogen and ground into powder using pestles. The total RNA was extracted by total RNA extraction reagent (TRIzol; Ambion, USA) according to the manufacturer's instruction.

A small part of the extracted RNA was removed for quantification and determination of purity at 230, 260, and 280 nm wavelengths by measuring the optical densities (ODs) and calculating the OD_260_/OD_280_ ratio. The OD_260_/OD_280_ ratios ranged from 1.8 to 2.0, demonstrating that the extracted RNA was of high purity. The remaining RNA samples were stored at −80°C for the following experiments.

To synthesize complementary DNA (cDNA), the 5 × TransScript All-in-One cDNA Synthesis SuperMix (Kangwei Co., China) for qPCR was used with reference to the manufacturer's instructions.  Table 1 shows the specific reverse transcription system used in this study. The reaction conditions of the complete reverse transcription system were as follows: reverse transcription at 42°C for 15 min, inactivation at 85°C for 5 min on Hamburg PCR Thermal Cycler (Eppendorf AG, 22331), and storage at 4°C for later usage.

All of the qRT-PCR primers for functional and internal reference genes were synthesized by Ningbo Zhenhai Baichuan Biotechnology Co., Ltd. (Zhejiang Province, China). Glyceraldehyde-3-phosphate dehydrogenase (GAPDH) was used as the internal reference to detect the relative mRNA expression levels of *α*-SMA and TGF-*β*1 in tissue samples on days 7, 14, and 28.  Table 2 shows the specific sequences of the primers used in this study. cDNA samples were used as templates for qRT-PCR. TransStart Tip Green qPCR SuperMix (TransGen Biotech Co., China) was used to measure the expression of the target gene after each polymerase chain reaction cycle.  Table 3 shows the specific qRT-PCR system, and the reaction was performed according to the following conditions: 94°C predenaturation for 30 s; 40 cycles of 94°C denaturation for 5 s, 62°C annealing for 15 s, and 72°C extension for 10 s.

### 2.6. Statistical Analysis

Data are expressed as the mean ± standard deviation. Statistical analysis was conducted by one-way analysis of variance using SPSS (SPSS 22.0; IBM, Armonk, NY, USA). *P* values <0.05 were considered to be statistically significant.

## 3. Results

### 3.1. Diabetic Model

**Figure 2:**
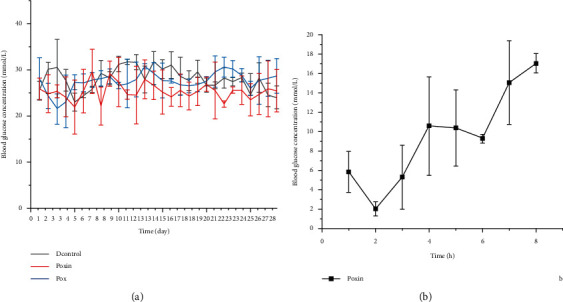
Mean (±SE) plasma glucose profiles of DControl, Pox, and Poxin groups for the 28 days of treatment period (a). Insulin injections were given in the morning and night, twice a day, with a dosage of 5 IU/kg/d. Their blood glucose could be maintained below 16.7 mmol/L for about 7 hours after injection (b).

The postprandial blood glucose concentrations of the DControl group, Pox group, and Poxin group are shown in Figure 2(a). The blood glucose concentration of all groups was maintained at >16.7 mmol/L, indicating successful establishment of the diabetic mouse model. After the mice in the Poxin group were injected with insulin, their blood glucose levels were maintained <16.7 mmol/L for approximately 7 h (Figure 2(b)). The overall blood sugar level of the Poxin group was slightly lower than that of the DControl group and Pox group, likely due to the insulin injection (Figure 2(a)).

### 3.2. Wound Closure

**Figure 3:**
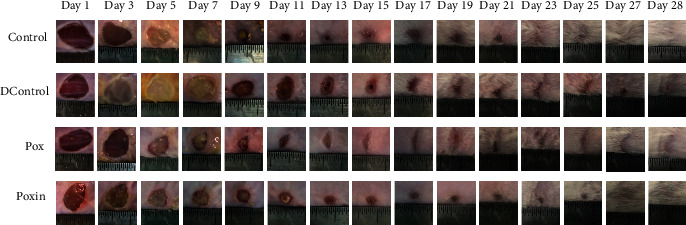
Wound closure of animals in Control, DControl, Pox, and Poxin. Wound closure was documented with a digital camera at a fixed distance every other day from day 1 to day 28 of the operation in all study groups.

**Figure 4:**
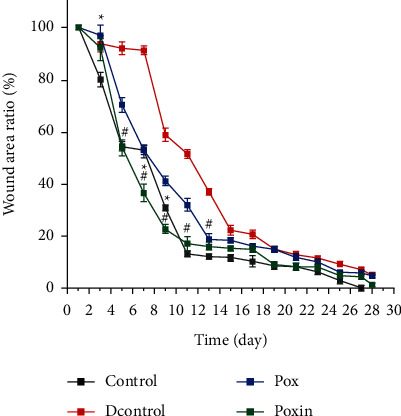
Percent of closure is shown on the graphic. Data were expressed as the mean ± SE of three mice per group. ^*∗*^*P* < 0.05, the Poxin group vs. the Control group. ^#^*P* < 0.05, the Poxin group vs. the DControl group.

Full-thickness incisional wounds were surgically created on the backs of mice, and the wound closure was monitored (Figure 3). The speed of wound healing varied according to the treatment (Figure 4). On the 7th day, the wound area ratio of the Pox group was 53.11%, which was significantly smaller than that of the DControl group (*P* < 0.05). On the 15th day, the wound area ratio of the Pox vs. the DControl group was 18.49% and 22.23% (*P* < 0.05). On the 28th day, the wound area ratio of the Pox vs. the DControl group was 4.85% and 5.22% (*P* > 0.05). The wound area ratio of the Pox group was lower than that of the DControl group during the first 28 days, implying that mice in the Pox group recovered faster than those in the DControl group. The wound healing speed of the Poxin group was improved compared to the Pox group. On 7, 14, and 28 days, the wound area ratio of the Poxin group was 36.57%, 15.2%, and 1.39%, respectively, which was smaller than that of the Pox group (*P* < 0.05). Thus, poloxamer plus insulin enhanced wound healing in this model.

### 3.3. Histologic Examination

**Figure 5:**
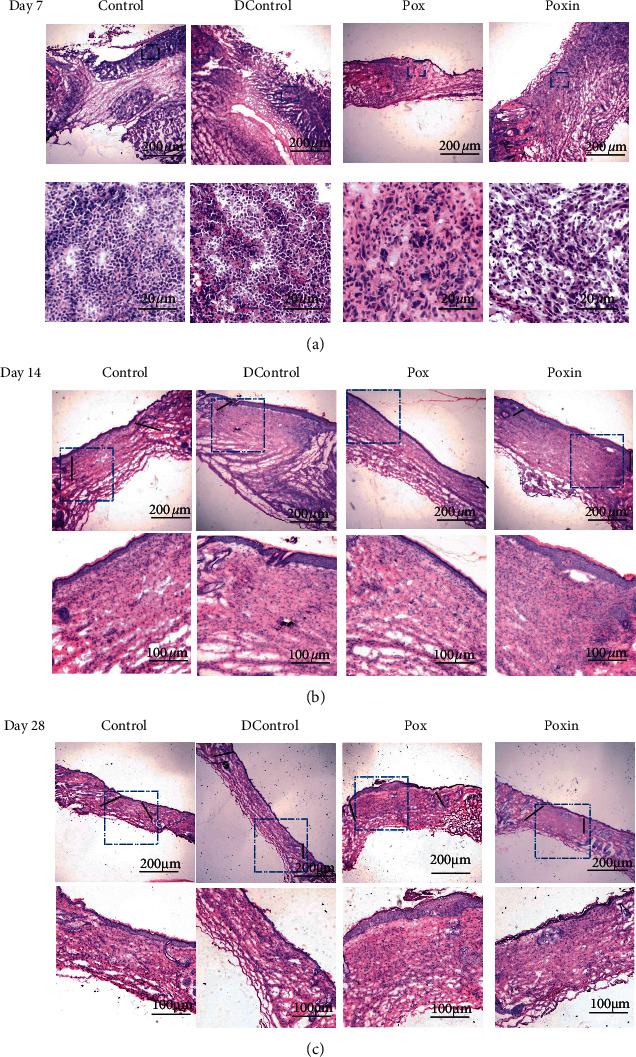
Histological photographs of a full-thickness nonsutured skin wound: Control, DControl, Pox, and Poxin groups at days 7, 14, and 28 after operation, respectively. Below figures are the magnifications of square zone on the up figures at the same day time. The black lines point the wound margin.

An overview specific to wound healing was observed in all histological specimens of all groups (Figure 5). On the 7th day, the healthy control group and Pox group demonstrated vast amounts of inflammatory cell infiltration (Figure 5(a), blue dots) to resist bacterial infection of the wound. On the 14th day, a single layer of epithelium began to appear on the granulation tissue of each group, which was mainly due to the proliferation of basal cells around the wound, and slowly migrated to the center of the wound. The wound area of the Control and Poxin groups (Figure 5(b), the area between two black lines) was significantly smaller than that of the other two groups. On the 28th day, the wounds in the Control group and Poxin group (Figure 5(c), the area between the two black lines) healed faster, the wound area was smaller, and more skin appendages appeared compared with the other two groups (Figure 5(c)).

**Figure 6:**
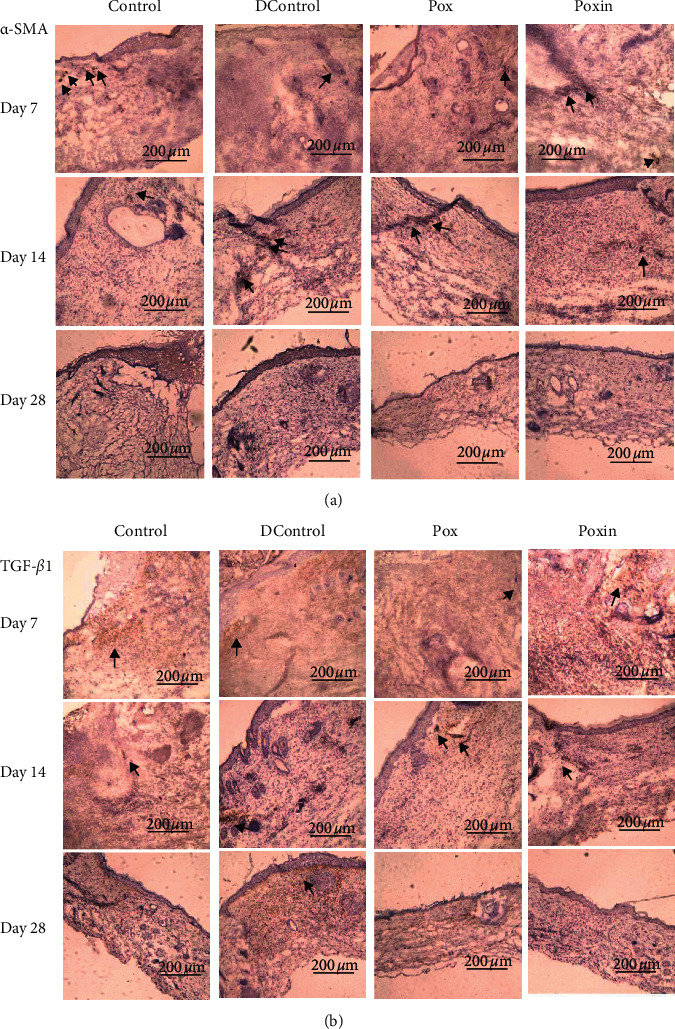
*α*-SMA and TGF-*β*1 antigen expression in the wound bed on 7, 14, and 28 days after wounding: samples, DControl, Pox, and Poxin groups. Black arrows gave the examples about secretion sites of the antigens.

Immunohistochemical analyses of *α*-SMA and TGF-*β*1 in each animal group are shown in Figure 6.*α*-SMA, a marker of myofibroblast differentiation, expressed increasingly in the Poxin and Control groups (brown color pointed by black arrows), compared with the other two groups on the 7th day, whilst the contrary result was observed on the 14th day. After that, expression of *α*-SMA was almost invisible in all samples, for example, at day 28. The expression of TGF-*β*1 in the entire wound area of the Poxin and Control groups on the 7th day was significantly higher than that in the other two groups, but it was lower than the other two groups on the 14th day. On the 28th day, the expression of TGF-*β*1 was negligible, except for the animals in the DControl group.

### 3.4. qRT-PCR for *α*-SMA and TGF-*β*1

**Figure 7:**
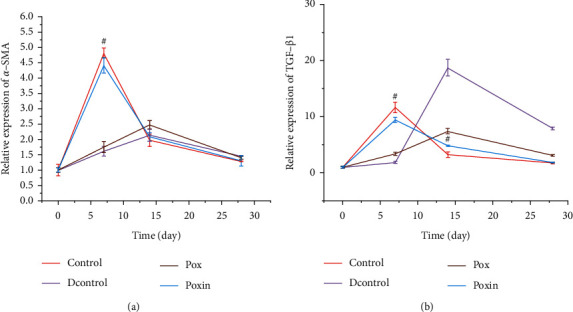
qRT-PCR quantification of the mRNA expression of *α*-SMA (a) and TGF-*β*1 (b) at different time points (days 7, 14, and 28) after wounding. Data were standardized by the expression level of GAPDH in each sample and presented as the relative expression of those from each group. ^*∗*^*P* < 0.05, the Poxin group vs. the Control group. ^#^*P* < 0.05, the Poxin group vs. the DControl group.

qRT-PCR was performed to measure the relative expression levels of *α*-SMA and TGF-*β*1 in wound tissues on the 7th, 14th, and 28th day of intervention (Figures 7(a) and 7(b)). The relative mRNA expression levels of *α*-SMA are shown in Figure 7(a). On the 7th day, *α*-SMA expression in the Poxin group was similar to that in the Control group (4.42 ± 0.25 vs. 4.8 ± 0.16, *P* > 0.05) but was significantly higher than that in the DControl and Pox groups (4.42 ± 0.25 vs. 1.6 ± 0.16, *P* < 0.05; 4.42 ± 0.25 vs. 1.76 ± 0.18, *P* < 0.05). On the 14th day, the Poxin group showed a significant downregulation of *α*-SMA production compared with that on day 7 (2.08 ± 0.13 vs. 4.42 ± 0.25, *P* < 0.001), in the DControl and Pox mice (2.08 ± 0.13 vs. 2.14 ± 0.20, *P* > 0.05; 2.08 ± 0.13 vs. 2.48 ± 0.14, *P* < 0.05).

Similar results were observed when the transcription of TGF-*β*1 mRNA was analyzed (Figure 7(b)). On the 7th day, the Poxin group displayed a significant increase in TGF-*β*1 mRNA compared to that of the DControl and Pox groups (9.45 ± 0.42 vs. 1.85 ± 0.15, *P* < 0.05; 9.45 ± 0.42 vs. 3.37 ± 0.27, *P* < 0.001), which is similar to the normal mice (9.45 ± 0.42 vs. 11.69 ± 0.95, *P* > 0.05). On the 14th day, Poxin-treated mice showed a significant decrease in TGF-*β*1 production compared to that on day 7 (*P* < 0.001). The same trend was observed compared to the Control and Pox groups (4.83 ± 0.21 vs. 18.72 ± 1.48, *P* < 0.05; 4.83 ± 0.21 vs. 7.46 ± 0.47, *P* < 0.05).

## 4. Discussion

As damaged skin can easily lead to infection, ulceration, and even limb necrosis, there is an urgent need to heal wounds quickly and effectively [25]. For patients with diabetes, impaired angiogenesis, macrophage deficits, and dysfunction retard all stages of wound repair [26]. Literature [27, 28] revealed that application of topical insulin chitosan nanoparticles improved wound healing in diabetic humans and animals. However, the major challenges for use of topical insulin are molecular instability and sustained delivery effect. In our previous work, Poloxamer 407 was used as hydrogel-like substrate, in which antibacterial guanidine-based polymers were loaded to assist the repair of defected animal skin [18]. Poloxamer 407 was verified to have good biocompatibility, though its biofunction in promoting wound healing was insufficient. Therefore, we developed a novel treatment, poloxamer hydrogel combined with insulin injection, in this work. The efficiency and the mechanism of systematic insulin injection plus topical hydrogel have not been fully investigated.

The diabetic animals presented a reduced wound healing rate due to the lack of energy materials in the body, leading to the loss or lack of recruitment of active cells in the wound [29]. The application of only poloxamer hydrogel on the top of wounds did not exhibit an advantage in sealing rate of skin. However, the combination of poloxamer and insulin increased skin healing rates, including a reduction in tissue cavity area and an increase in tissue density. We believe that there is a dose-dependent response of wound. Once we preliminary screened in insulin dosage from 1.0 to 5.0 IU/kg/d, the wound healing rate of the Poxin group promoted stably at the dosage of 5.0 IU/kg/d compared with that of the Pox group. Thus, we took this dosage as the parameter in this work. However, we should always be cautious because it is possible to introduce insulin resistance if we give additional amount of drug, which will increase proinflammatory cytokines, endoplasmic reticulum stress, and cell death during the recovery period [30]. Thus, we will conduct more research in future about this issue, the correlation between insulin dosage and diabetic wound closure.

The mRNA levels of TGF-*β*1 and *α*-SMA were examined to further investigate the underlying mechanism of this treatment. At the early stage, TGF-*β*1 mRNA levels were significantly higher in the Control group and Poxin group, compared to the DControl group and Pox group, respectively. Increased TGF-*β*1 mRNA indicates increased TGF-*β*1 protein expression, which in turn contributes to fibroblast recruitment to promote wound healing. Understanding the mechanisms of how insulin improves diabetic wound healing is important because it will provide insight into potential manipulation of the impaired healing process. However, the role of insulin in skin physiology is only partially known. Insulin can activate cytokines to help recover the wound [31]. Through metabolism and synthesis activities, insulin also plays an important role in cell differentiation and survival. Insulin receptors are expressed in keratinocytes and can be stimulated by insulin to activate intracellular signaling pathways [32]. We have previously shown that insulin stimulates the proliferation of mouse keratinocytes [33] and the production of matrix proteins, including fibronectin, collagen, and various proteoglycans [34, 35]. Insulin also reduces high-glucose-induced macrophage polarization to an M1 phenotype and promotes the transition from an M1 to M2 phenotype [20]. TGF-*β*1, secreted by macrophages, stimulates the expression of integrin subunits, which promote keratinocyte migration on the provisional extracellular matrix [36]. At the later proliferative stage, fibroblasts activated by TGF-*β*1 differentiate into myofibroblasts, which promote contraction, re-epithelialization, and closure of the wound [22]. We found that insulin-treated mice had high TGF-*β*1 mRNA levels, similar to the normal mice at an early stage, but decreased at the later stage. We therefore suggest that TGF-*β*1 plays an important role in the insulin-accelerated wound healing process, apart from the abovementioned mechanism. At the inflammatory stage, insulin treatment recruited macrophages to the unwounded skin margin and agminated macrophages exerted their roles in wound healing, including the induction of TGF-*β*1 secretion, which possibly induces fibroblast to myofibroblast transition. Myofibroblasts express a highly characteristic protein *α*-SMA, which is responsible for wound contraction [37].

TGF-*β*1 also plays a role in the formation of granulation tissue [38], subsequent re-epithelization, proliferation of fibroblasts and keratinocytes, and collagen formation during the granulation phase [39], as well as wound closure and scar formation during the maturation phase [40]. Skin fibrosis is characterized by disorganized collagen formation [41]and epidermal architecture [42]. From previous studies, pathologic fibrosis and scarring occur mostly because of the persistent presence of TGF-*β*1 [43]. Continual expression of *α*-SMA in fibroblasts may result in deposition of extracellular matrix and scar contracture [44]. In our study, enhancement of TGF-*β*1 expression was observed on day 7 in insulin plus poloxamer treated animals (Poxin group). Insulin appears to increase the release of TGF-*β*1, which promotes the migration and proliferation of fibroblasts and the transition from fibroblasts to myofibroblasts, resulting in increased collagen synthesis at an early stage. Therefore, we hypothesize that insulin treatment upregulates *α*-SMA expression by inducing TGF-*β*1 at an early stage of wound healing, leading to the recruitment of macrophages and the transformation of fibroblasts to myofibroblasts. The mRNA expression of *α*-SMA and TGF-*β*1 in normal animals was elevated on day 14 (granulation phase), which contributes to the formation of skin fibrosis.

In summary, we discovered that poloxamer alone was ineffective in the context of wound healing, while poloxamer plus insulin treated mice displayed enhanced wound healing. Upon further investigation of the mechanism, we found that the expression of TGF-*β*1 and *α*-SMA was increased significantly during the early phase of diabetic wounds, which correlated with early wound closure. The increase in TGF-*β*1 expression after insulin injection promoted the wound healing progress, suggesting that TGF-*β*1 plays an important role in insulin-accelerated wound healing. These findings are both interesting and valuable for the future design of chronic wound treatment in the clinic.

## Figures and Tables

**Table 1:** Total RNA reverse transcription system.

Component	Volume (*μ*L)	Final concentration
dNTP mix, 2.5 mM	4	500 *μ*M
Primer mix	2	—
RNA template	X	50 pg–5 *μ*g
5×RT buffer	4	1×
DTT, 0.1 M	2	10 mM
HiFiScript, 200 U/*μ*L	1	—
RNase-free water	Up to 20	—

**Table 2:** Primer sequences for quantitative real-time PCR.

Gene	Primer sequence (5′–3′)
GAPDH	Forward: 5′-GACAGGATTGACAGATTGATAGC-3′
	Reverse: 5′-AGCATGCCAGAGTCTCGTT-3′

*α*-SMA	Forward: 5′-GTCCCAGACATCAGGGAGTAA-3′
	Reverse: 5′-TCGGATACTTCAGCGTCAGGA-3′

TGF-*β*1	Forward: 5′-CCACCTGCAAGACCATCGAC-3′
	Reverse: 5′-CTGGCGAGCCTTAGTTTGGAC-3′

**Table 3:** Real-time fluorescence-based quantitative PCR (qRT-PCR) system.

Reagent	Volume (*μ*L)
2 × TransStart Tip Green qPCR SuperMix	10
Forward primer (10 *μ*M)	0.4
Reverse primer (10 *μ*M)	0.4
ddH_2_O	7.2
cDNA	2
Total volume	20

## Data Availability

The data used in this study are available upon request to the corresponding author.
